# Flexible Flaps Inspired by Avian Feathers Can Enhance Aerodynamic Robustness in low Reynolds Number Airfoils

**DOI:** 10.3389/fbioe.2021.612182

**Published:** 2021-05-07

**Authors:** Yuta Murayama, Toshiyuki Nakata, Hao Liu

**Affiliations:** ^1^Graduate School of Science and Engineering, Chiba University, Chiba, Japan; ^2^Graduate School of Engineering, Chiba University, Chiba, Japan

**Keywords:** biomimetics, drone, birds, feather, wing, aerodynamics, flexibility, fluid-structure interaction

## Abstract

Unlike rigid rotors of drones, bird wings are composed of flexible feathers that can passively deform while achieving remarkable aerodynamic robustness in response to wind gusts. In this study, we conduct an experimental study on the effects of the flexible flaps inspired by the covert of bird wings on aerodynamic characteristics of fixed-wings in disturbances. Through force measurements and flow visualization in a low-speed wind tunnel, it is found that the flexible flaps can suppress the large-scale vortex shedding and hence reduce the fluctuations of aerodynamic forces in a disturbed flow behind an oscillating plate. Our results demonstrate that the stiffness of the flaps strongly affects the aerodynamic performance, and the force fluctuations are observed to be reduced when the deformation synchronizes with the strong vortex generation. The results point out that the simple attachment of the flexible flaps on the upper surface of the wing is an effective method, providing a novel biomimetic design to improve the aerodynamic robustness of small-scale drones with fixed-wings operating in unpredictable aerial environments.

## Introduction

As unmanned aerial vehicles, called drones, have been used for various tasks recently (Floreano and Wood, 2015; Liu et al., 2016), it has been increasingly more important to improve their flight performance, such as stability and efficiency especially when they fly in urban areas. The drones tend to become unstable under the unpredictable wind that is commonly observed in natural environments. The perturbations in the attitude must be fixed as quickly as possible in order to stay airborne even though the disturbances are difficult to predict.

In order to deal with these challenges, engineers have often been inspired by the functions of flying animals in nature (Bechert et al., 2000; Chin et al., 2017; Luca et al., 2017).

Several strategies have been proposed to improve the capabilities of current drones, inspired by animal flight control studies on flying insects, birds, bats, and other animals (Franceschini et al., 2007; Lentink, 2014). This research approach, called biomimetics, has an impact not only on drone-related research topics but also on a variety of research fields such as robotics and bioengineering (Lepora et al., 2013). It is expected that biomimetics will play an essential role in the development of new technologies that have social significance in the future (Lepora et al., 2013). Besides, biomimetic robots have also been used as a model to study living organisms (Romano et al., 2019a), contributing to developing a mixed field of engineering and biology (Romano et al., 2019b).

**FIGURE 1:**
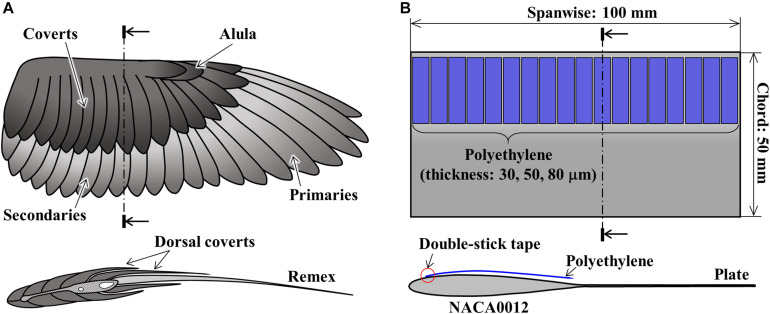
Schematic diagram of panel **(A)** a bird wing and a cross-section and **(B)** the bird-inspired wing model.

Birds are frequently selected as sources of inspiration because birds are similar in size (i.e., Reynolds numbers) to drones and have excellent flight capabilities. It is known that avian wings have various features in their structural design (Figure 1A) and flight techniques that make them seem different from aerial vehicles with rigid wings: for example, flexible muscles, feather transmissibility, and flexibility (e.g., Brown and Fedde, 1993; Müller and Patone, 1998; Reynolds et al., 2014). Previous studies showed that these characteristics contribute to the improvement of bird flight performance. For example, it is known that the separated wingtip slots reduce the induced drag (Tucker, 1995), and the small feather called alula near the leading-edge delays stall at a high angle of attack (Álvarez et al., 2001; Lee et al., 2015). Attentions are also paid to the role of flexible feathers that deform during a flight (Carruthers et al., 2007; Cleaver et al., 2014). Experimental and computational studies on the wings with additional flap inspired by covert feathers have shown that passively pop-up flap enhances the lift force and improve efficiency. The studies shown above have been performed mainly under the assumption of uniform flow (Kernstine et al., 2008; Schlüter, 2009; Rosti et al., 2017). Drones are, however, expected to operate at the atmospheric boundary layer where the various unsteady wind is generated due to the friction of the wind and the ground (Watkins et al., 2006). For drones, the improvement of flight stability under unsteady and unpredictable wind disturbances (e.g., gusts and eddy currents) is as significant as improvement of efficiency.

In this study, aiming at the development of a highly robust wing for drones against disturbances, an experimental wing with covert-inspired flexible flaps near the leading edge of the upper surface was fabricated. With a specific focus on the effect of flexible flaps on the robustness of the wing in disturbance flow, three types of flexible flaps with different stiffnesses and, for the comparison, baseline fixed-wing without attachment were tested by wind tunnel experiments. To clarify the mechanism behind the effect of flexible flaps, we further combined the flow visualizations by using particle image velocimetry.

## Materials and Methods

### Design of Flexible Feathered Wing

The experimental wing model (Figure 1B) is inspired by the avian wing (Figure 1A). Avian wings are covered with several types of feathers that arise from the skin and bones of the leading-edge, and thin plate-like remiges grow toward the trailing-edge. While birds can actively and passively control the camber of their wing (Videler, 2005), the experimental model in this study has no camber for simplicity.

The wing model is composed of NACA0012 airfoil with an extended trailing-edge plate and flexible flaps. The basic form of the wing was made by cutting the aluminum plate (A5052) with a CNC cutting machine (MDX-540, Roland DG Corporation). The chordwise and spanwise lengths of the wing were 50 and 100 mm, respectively. After several films with different lengths and widths were tested, rectangular low-density polyethylene films with a length of 20 mm and a width of 5 mm were selected for this study. Accordingly, the chordwise stiffness of the film was much lower than the spanwise stiffness. Eighteen flaps were fixed to the upper surface of the wing by a double-stick tape at 2.5 mm (5% chord) from leading-edge like a cantilever.

In this study, three models with flexible flaps with 30, 50, and 80 μm-thickness were tested compared with basic wing without flexible flaps. Flexible flaps of different thicknesses were utilized to see the effect of stiffness without changing their geometric size. We call the models with 30, 50, and 80 μm flaps model-30, model-50, and model-80, respectively. The model without flaps is called basic wings.

### Wind Tunnel Experiment

Experiments were conducted in a low-speed wind tunnel at Chiba University (Ikeda et al., 2018). The test section of the wind tunnel is 2 m-long with a cross-section of 1 × 1 m. The side walls are made of transparent acrylic boards. In this work, experiments of force measurement and particle image velocimetry were performed at wind speed U = 5 ms^–1^. In this study, with the wing chord to be a reference for the length scale and the wind speed to be a reference for the velocity scale, the Reynolds number is about 16,000.

#### Aerodynamic Force Measurement

**FIGURE 2:**
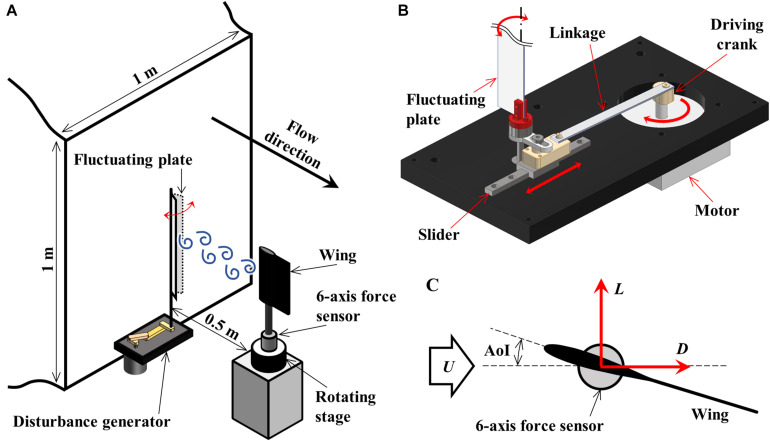
**(A)** Experimental setup for aerodynamic force measurements in the low-speed wind tunnel. The fluctuation generator was mounted upstream for measurements in disturbed flow. **(B)** The fluctuation plate was driven by a motor via a slider-crank mechanism. **(C)** Lift, L, and Drag, D, were measured by the 6-axis force sensor.

Figure 2 is a schematic diagram of the setup for the aerodynamic force measurements. As shown in Figure 2A, the wing was mounted vertically to a 6-axis force sensor (Nano17Ti, ATI Industrial Automation) via a 3D-printed sting with the quarter chord of the wing and center of the force sensor aligned.

The force sensor was fixed on a rotating stage (SGSP-80YAW, SIGMAKOKI Co., Ltd.), and thus angle of incidence (AoI) of the wing was varied. The rotating stage was operated remotely by a stage controller (SHOT-702, SIGMAKOKI Co., Ltd) with an accuracy of 0.15 degrees. The aerodynamic forces on the wing were dynamically measured by the force sensor and were digitalized by an A/D converter (USB-6210, National Instruments Corp.) with a sampling rate of 1 kHz.

The aerodynamic forces were measured in two experimental conditions: in uniform flow and in disturbed flow. Measurements in uniform flow were performed twice in the range of AoI 0–20 degrees (1 degree increments) with a sampling time of 10 seconds. For the experiments in disturbed flow, the disturbance generator was additionally mounted to the wind tunnel at 0.5 m upstream of the wing. The disturbance generator (Figure 2B) consists of a motor-driven slider-crank mechanism with a rigid plate made of aluminum. The frequencies of the flow fluctuation were altered by controlling the rotating speed of the motor. In this research, the frequencies of the flow fluctuation are set in the range of 2–25 Hz (1 Hz increments), and measurements were performed twice with a sampling time of 30 s at AoI of 5 degrees. The measured forces at the force sensor were transformed into the lift, *L*, and drag, *D*, based on the AoI (Figure 2C). *L*, *D*, and non-dimensional lift and drag coefficients (*C*_*L*_, *C*_*D*_) were computed by dividing the force components by 0.5*rU*^2^*S*,

(1) $$\begin{array}{c} \left\{ \begin{array}{c} L=F\,x\,\cos\text{AoI}-F\,y\,\sin\text{AoI},\\ D=F\,x\,\sin\text{AoI}-F\,y\,\cos\text{AoI},\\ C_{L}=\frac{2\,L}{\rho \,U^{2}\,S},\\ C_{D}=\frac{2\,D}{\rho \,U^{2}\,S}, \end{array}\right. \end{array}$$

where ρ is the air density, *U* is the wind velocity, *S* is the projected wing area. These aerodynamic forces dynamically changed with time, especially in disturbed flow. Therefore, the robustness of the wing was evaluated by using the standard deviation (SD) of the aerodynamic force across the sampling time (10 s or 30 s), which was defined by the following equation,

(2) $$\begin{array}{c} \text{SD}=\sqrt{\frac{1}{n}\sum_{i=1}^{n}{\left(d_{i}-\bar{d}\right)}^{2},} \end{array}$$

where $\bar{d}$ is the average of *n* data.

#### Particle Image Velocimetry (PIV)

**FIGURE 3:**
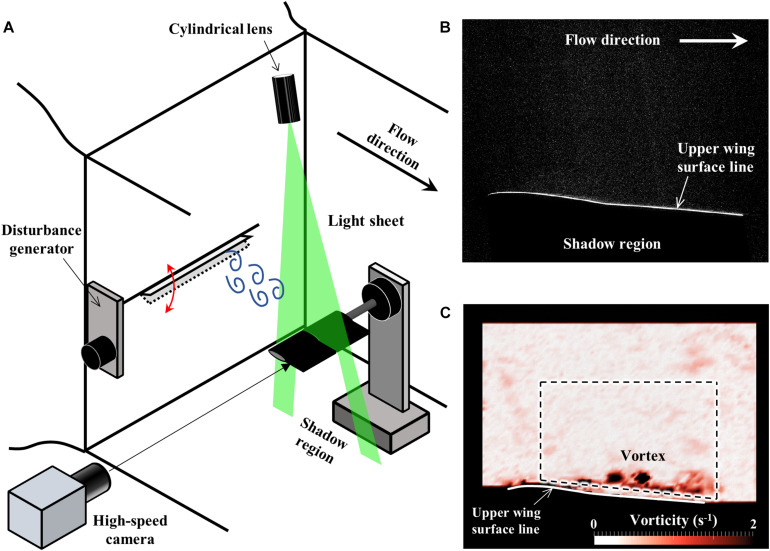
**(A)** Schematic diagram of the experimental setup for 2D-PIV measurements. **(B)** A raw image of PIV measurements. Since the laser light was irradiated from above of the wing, the area lower than the wing was in the shadows and too dark to see the particles. **(C)** The sample flow field is visualized by the distribution of the vorticity. The surrounding area in black was masked during the analysis of the PIV due to the low light level. The area inside the dashed line was defined to calculate the ratio of high vorticity area.

The PIV measurement system (Figure 3A) consists of an Nd:YAG pulsed laser (LDP-100MQG, Lee Laser, Inc.), a timing controller (LC880, SEIKA Digital Image Corporation), a seeding generator (PivPart14, PivTec GmbH), and a high-speed camera (FASTCAM SA3, PHOTRON LIMITED) with an optical lens (150 mm, SIGMA Corporation). The wing assembly was mounted horizontally to sting, unlike the force measurement. A light sheet generated by a pulsed laser via a cylindrical lens was positioned above the wing to illuminate a mid-span streamwise plane, and images were recorded by the camera positioned to the side. The exposure timing of the laser and camera was synchronized through the timing controller. The PIV images were acquired at a rate of 250 pairs per second for a total time of two seconds. The resolution of the image sensor was 1024 × 1024 pixels, and the field of view was about 72 mm × 72 mm. The PIV images (Figure 3B) were analyzed using commercial PIV software (Koncerto II, SEIKA Digital Image Corporation).

The interrogation window size of 24 × 24 pixels with 12 pixels step size was selected to generate a flow vector field. The lower wing regions which were shadowed by the wing were masked, and thus upper wing regions were used for PIV analysis. In order to evaluate the effect of the flexible flaps on the flow field, the vorticity (Ω), calculated from the flow vector, was defined by the following equation,

(3) $$\begin{array}{c} \Omega =\frac{\partial v}{\partial x}-\frac{\partial u}{\partial y}, \end{array}$$

where *u* is the velocity component along the stream (*x*-direction) and *v* is the velocity component perpendicular to the stream (*y*-direction). The ratio of the area where the vorticity was larger than a threshold (Ω > 1.0 s^–1^) was calculated from the vector field. The area defined for comparing the vorticity was inside the fixed region shown by the dashed lines in Figure 3C. The defined area excludes the vorticity of the wing surface in order to assess only the vortices that are detached from the wing.

The raw images of the PIV measurements contained not only the particles but also the illuminated flexible flap. Therefore, the images were also used to measure the deformation of the flexible flap. The trajectory of the tip of the flexible flap was tracked using commercial software (MATLAB, The MathWorks, Inc.).

## Results

### Aerodynamic Performance

**FIGURE 4:**
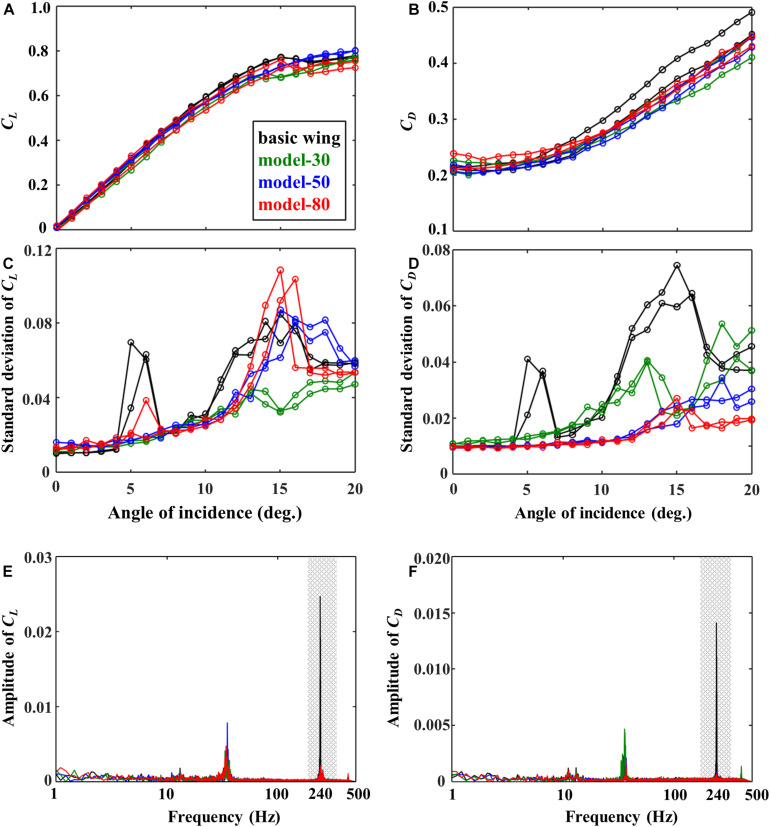
**(A)** Mean lift and **(B)** drag coefficients (*C*_*L*_, *C*_*D*_), the standard deviations of panel **(C)** lift and **(D)** drag coefficients, and the frequency spectra of panel **(E)** lift and **(F)** drag coefficients at an angle of incidence of 6 degrees.

Figures 4A,B shows the result for lift and drag coefficients of the wings at each AoI in uniform flow. While the difference in the lift coefficient was small among all models, the lift coefficient curve of the model-50 showed a smoother stall behavior than the other models. Additionally, it was found that the drag coefficient decreases in the models with flexible flaps compared with the basic wing, especially when AoI was greater than 10 degrees. The standard deviations of lift and drag coefficients (Figures 4C,D) were smaller in the models with flexible flaps than in the basic wing at AoI around 6 degrees. By comparing the frequency spectra of the standard deviation of lift and drag coefficient at AoI of 6 degrees (Figures 4E,F), it was found that the basic wing showed a larger peek at high frequency (around 240 Hz) than the other models with flexible flaps. The force fluctuations in the basic wing are thought to be due to the laminar separation (Kim and Chang, 2010), which induces the vibration of the trailing-edge plate. Besides, it can be seen that the variation of the model-30 was smaller than the other models at AoI around 15 degrees.

**FIGURE 5:**
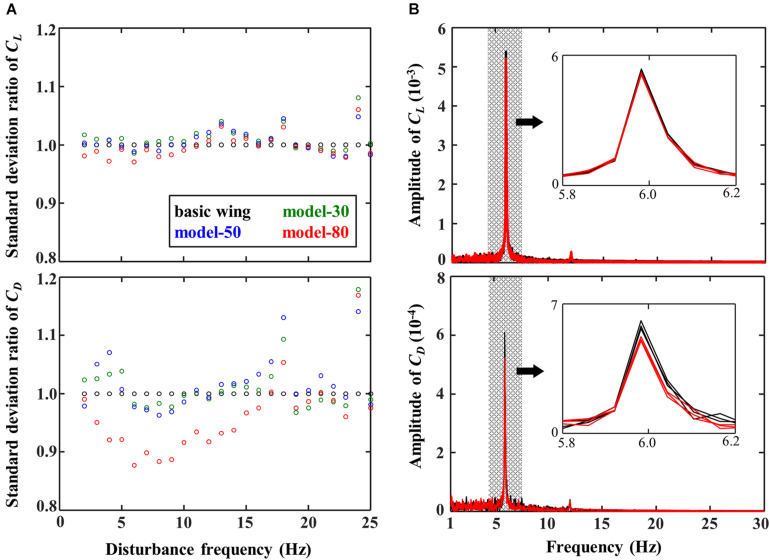
**(A)** Effect of the disturbance frequencies on the standard deviation ratio of *C*_*L*_ and *C*_*D*_ relative to the basic wing at an angle of incidence of 5 degrees. **(B)** Frequency spectra of *C*_*L*_ and *C*_*D*_ at disturbance frequency of 6 Hz.

Figure 5A shows the effect of the flexible flaps on the standard deviation of the lift and drag coefficient divided by those of the basic wing at various disturbance frequencies. These results were obtained after filtering the raw data by a third-order low-pass Butterworth filter with a cut-off frequency of 1.5 times the disturbance frequency in order to reduce the effect of the wing and force sensor resonance (about 36 Hz) contained in the raw data. With reference to the basic wing, the differences of the standard deviation were clearer for the drag coefficient than those for the lift coefficient. The model-80 reduced the standard deviation in the drag by about 10 % when the disturbance frequency was around 6–9 Hz. Similarly, the comparison of the frequency spectra for a disturbance frequency of 6 Hz (Figure 5B) shows that the model-80 reduces the disturbance frequency peak compared with the basic wing, especially in the case of the drag coefficient.

### Flow Visualization

**FIGURE 6:**
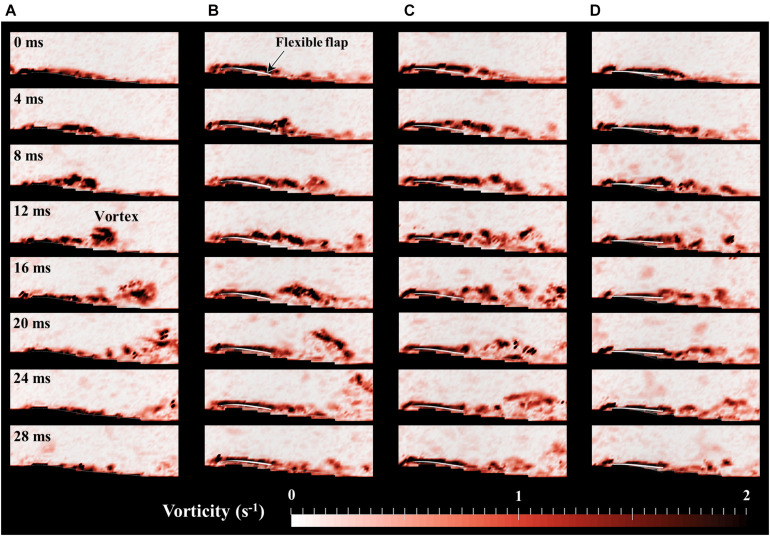
Time-series of the vorticity field within 28 ms around **(A)** basic wing, **(B)** model-30, **(C)** model-50, and **(D)** model-80 at disturbance frequency of 6 Hz. The ratio of high vorticity area is minimized when *t* = 0 ms in each model.

Figure 6 shows a time-series of the vorticity distribution near the wings in the disturbance of 6 Hz, where the standard deviation of the drag coefficient in the model-80 was reduced in force measurements (Figure 5A). The vortices were separated and moved toward the trailing-edge of the wing (8–24 ms in Figure 6A). While the large vortex is generated due to the separation on the basic wing (12–20 ms in Figure 6A), the model-80 with the flexible flap generated smaller vortices shedding (12–20 ms in Figure 6D). It was also observed that the flexible flap was deformed in response to the timing of the vortex. The negative pressure of the vortex presumably induced the deformation of the flexible flap.

**FIGURE 7:**
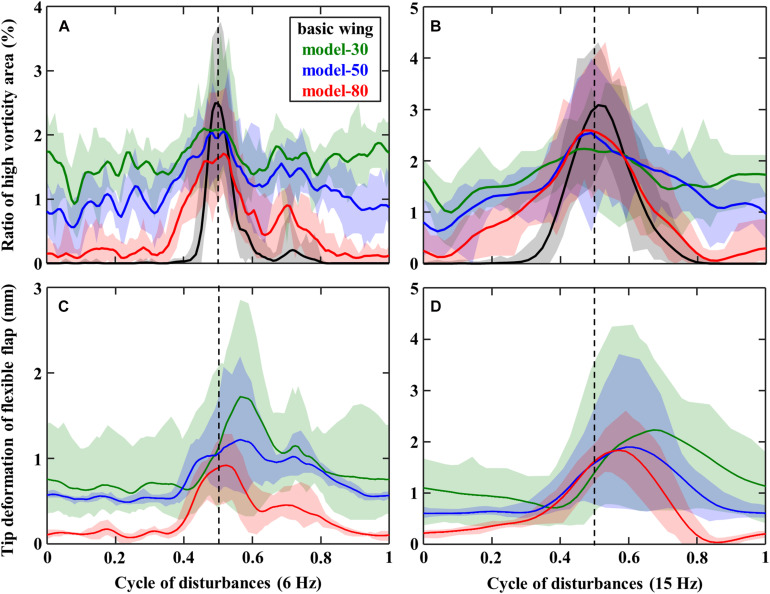
Time-series of panels **(A,B)** the ratio of high vorticity area and panels **(C,D)** the deflection of the tip of flexible flaps in the disturbance of panels **(A,C)** 6 Hz or **(B,D)** 15 Hz.

The time series of the high vorticity area defined in Figure 3C and the tip deflections of the flexible flaps obtained from images are summarized in Figure 7. The model-80 shows a smaller vorticity than other models with a flexible flap and a smaller peek than the basic wing at a disturbance of 6 Hz (Figure 7A). Similar results were obtained at disturbance of 15 Hz (Figure 7B), but the differences between the models were relatively smaller than those at 6 Hz. The time-series of the tip deflection of flaps in Figures 7C,D reveals that the deformation of the flexible flap in each model corresponds to its stiffness; the flap deflection is larger in the less stiff model. With the disturbance of 6 Hz, the timing of the maximum deformation of the model 80 was approximately matched with the timing of the maximum vorticity, but the timings of the other models were delayed from the timing of the vorticity peeks. Similarly, the deformation of the flexible flaps was delayed more with the less stiff flaps at a disturbance frequency of 15 Hz.

## Discussion

### The Effect of Flexible Flaps on Aerodynamic Performance

We found that the variation of the aerodynamic force on the wing in uniform flow can be reduced by attaching the flexible flaps near the leading-edge. It is suggested that the passive deflection of the flexible structure around the leading-edge suppressed the flow separation at the leading-edge, and thus did not induce high-frequency vibration (Figures 4C,D). The birds’ feathers are observed to deflect at a high angle of attack when the flow is thought to be highly unsteady (Carruthers et al., 2007). Therefore, it is reasonable to assume that the dorsal coverts of birds near the leading-edge have a similar function for the suppression of the flow separation in a uniform flow. This effect is comparable to the function of alula, which delays flow separation by generating longitudinal vortices (Lee et al., 2015), while the results in this study pointed out that the passive deformation of the feathers can suppress the flow separations.

The flexible flaps on the upper surface of the airfoil enhanced lift in previous studies (e.g., Bechert et al., 2000; Schlüter, 2009; Traub and Jaybush, 2010), but the lift enhancement was not clearly observed in this study (Figure 4A). The difference is thought to be because the previous studies placed the flaps near the trailing-edge, while the flexible flaps were attached around the leading-edge in this study. Therefore, the flexible flaps may have multiple roles depending on the locations with respect to the wing chord.

The variation of the aerodynamic forces of the model-80 with flexible flaps are found to be decreased at disturbance frequencies of 6–9 Hz compared with the basic wing without flexible flaps (Figure 5A). The force reduction of the other models with flexible flaps was relatively smaller than the model-80, while the tip deflection of the flexible flap was the smallest in the model-80 (Figure 7C). Therefore, the reduction of the variations is thought to require the appropriate amount of the deformation of the flexible flaps in response to the disturbances (Figure 7C). From the flow visualization, we found that the vortex shedding of the model-80 was smaller than that of the basic wing (Figure 6), which is probably because the timing of the deformation of the model-80 flaps matches with the vortex separation (Figures 7A,C). Given that the response of the flaps is completely passive, the mass and the flexibility of the attached flaps should be appropriately designed depending on the disturbance frequency in order to reduce the force fluctuations under disturbances. The hierarchical structure and the taper toward the tip of the avian feathers (Sullivan et al., 2017) may be beneficial in the more complex, natural environments because the complex structures may be able to respond to the disturbances in a wider range of frequencies.

Inspired by flying animals, various studies have tried to improve the flight control capability of drones with active mechanisms (Lentink, 2014). The distinctive feature of this research, which is related to the development of biomimetic drones, is the improvement of the flight stability of drones by using only passive mechanisms. Furthermore, from the results, it may be hypothesized that birds utilize flexible feathers as a device to passively adapt to the complex and changing wind environment around them.

The applications of flexible flaps may not be limited only to wings for drones. For example, the reduction of aerodynamic force fluctuation is beneficial for the slender vertical structures that are continuously exposed to wind load fluctuation, which leads to mechanical fatigue and damage (Repetto and Solari, 2004). A large number of studies have been devoted to the aerodynamics over a bluff body such as cylinders, and rigid or flexible splitter plate in the wake of bluff bodies are known to control the vortex shedding (e.g., Akili et al., 2005; Shulka et al., 2013). Mazellier et al. (2012) showed that the mean drag force applied on a square cylinder was reduced by feather-inspired porous plates fitted on the sides of the square cylinder. As shown in this study, the interaction between the fluid and flexible flaps may also reduce the fluctuations of aerodynamic forces applied to the bluff bodies. Thus, the adaptive, flexible flaps on the structure surfaces can be suggested as a simple strategy to enhance the maintainability and reliability of structures exposed to various wind disturbances.

## Conclusion

In this study, the effect of flexible flaps inspired by avian covert feathers on the aerodynamic performance of a fixed-wing has been investigated experimentally with a specific focus on its robustness against disturbances. Experiments were carried out for a wing model with flexible flaps of different stiffnesses, operating at the Reynolds number of approximately 16,000 in a low-speed wind tunnel. The aerodynamic force measurements were conducted with a force sensor, and the PIV measurements were utilized to visualize the flow fields near the wing.

The force measurements revealed that the wing model with flexible flaps could considerably suppress the fluctuations of aerodynamic forces in both uniform and disturbed flows. The results correspond with a pronounced reduction in the magnitude of vorticity on the upper surface of the wing with flexible flaps. Such reduction in the variation of aerodynamic forces was further confirmed to be strongly dependent on the stiffness of the flaps, and thus, there likely exists an optimal stiffness of the flexible flaps capable of reducing the disturbance-induced fluctuations at some specific disturbance frequencies.

Our results indicate that the covert feathers near the leading-edge may work as a passive flow-control device to enhance the aerodynamic robustness under aerial disturbances. Given its simplicity, the feather-inspired attachment can be used as an effective method for improving the flight stability of small drones with fixed-wing working in an environment with various disturbances.

## Data Availability Statement

The raw data supporting the conclusions of this article will be made available by the authors, without undue reservation.

## Author Contributions

TN conceived the study with YM and HL. YM performed the experiments. YM and TN analyzed the data. All authors designed the experiments, wrote and contributed to the final version of the manuscript and approved the submission.

## Conflict of Interest

The authors declare that the research was conducted in the absence of any commercial or financial relationships that could be construed as a potential conflict of interest.
